# The global lake area, climate, and population dataset

**DOI:** 10.1038/s41597-020-0517-4

**Published:** 2020-06-11

**Authors:** Michael F. Meyer, Stephanie G. Labou, Alli N. Cramer, Matthew R. Brousil, Bradley T. Luff

**Affiliations:** 1 School of the Environment, Washington State University, Pullman, Washington 99164 USA; 2 Center for Environmental Research, Education, and Outreach, Washington State University, Pullman, Washington 99164 USA; 3 Present Address: University of California San Diego, La Jolla, California 92093 USA

**Keywords:** Limnology, Hydrology

## Abstract

An increasing population in conjunction with a changing climate necessitates a detailed understanding of water abundance at multiple spatial and temporal scales. Remote sensing has provided massive data volumes to track fluctuations in water quantity, yet contextualizing water abundance with other local, regional, and global trends remains challenging by often requiring large computational resources to combine multiple data sources into analytically-friendly formats. To bridge this gap and facilitate future freshwater research opportunities, we harmonized existing global datasets to create the Global Lake area, Climate, and Population (GLCP) dataset. The GLCP is a compilation of lake surface area for 1.42 + million lakes and reservoirs of at least 10 ha in size from 1995 to 2015 with co-located basin-level temperature, precipitation, and population data. The GLCP was created with FAIR (findable, accessible, interoperable, reusable) data principles in mind and retains unique identifiers from parent datasets to expedite interoperability. The GLCP offers critical data for basic and applied investigations of lake surface area and water quantity at local, regional, and global scales.

## Abstract (summary)

**Table:** 

Measurement(s)	area of open water • temperature of air • volume of hydrological precipitation • population
Technology Type(s)	digital curation
Factor Type(s)	year • geographic location
Sample Characteristic - Environment	lake

Machine-accessible metadata file describing the reported data: 10.6084/m9.figshare.12328214

## Background & Summary

Local, regional, and global patterns in freshwater availability are increasingly important in times of a changing climate and growing human population. At the global scale, tracking changes in water abundance is more feasible than ever before as a result of advances in remote sensing techniques. Lehner & Döll^1^ were among the first to compile a global water quantity dataset (i.e., Global Lake Water Dataset GLWD), which included lakes greater than 10 ha in size. Since then, several studies have incorporated diverse remote sensing, topographic, and *in situ* data to catalog inland water quantity. For instance, Verpooter *et al*.^2^ created the Global Water Bodies database (GLOWABO) of ~117 million lakes from satellite data; Richey *et al*.^3^ employed GRACE data to assess change in recharge for 37 of the world’s largest aquifers; and Pekel *et al*.^4^ used LANDSAT data to identify and quantify global surface water from 1984 to 2015. Remote sensing’s relatively high frequency and extensive temporal record has enabled comparisons of water quantity change within years and across decades^5–7^. Similarly, local-scale investigations of water abundance have incorporated remote sensing data to explain hydrological and biological phenomena. For example, Anderson *et al*.^8^ paired remote sensing data with stable isotope and climate data to create water budgets for high-latitude boreal lakes. At both local and global scales these analyses conclude that trends in water abundance are highly heterogeneous over space and time, with regional patterns being explained by differences in human demand, climate, the physical landscape, or interactions between these drivers.

Despite extensive data availability to address questions of water quantity at various scales, the pace of research is often limited by the amount of time and computational resources required to aggregate and harmonize disparate data sources. Likewise, managing and working with highly voluminous environmental data can hinder users without advanced computational skills and resources, such as knowledge of a coding language or access to high performance computers for “big” data analyses^9,10^. While recent efforts to capture global scale changes have created opportunities to address water quantity questions as they relate to human demand and climate variability, many researchers, especially those focused at local scales, may be limited by the computing infrastructure and researcher skill set required to integrate existing environmental datasets.

To help bridge the distance between available global water-related data sources and potential users with a range of computational capacities and experiences of managing large, disparate data sources, we integrated existing long-term datasets for lake locations and characteristics, water surface area, climate, and human population to create the unified Global Lake area, Climate, and Population (GLCP) dataset. The GLCP contains over 1.4 million lakes of at least 10 ha in surface area, with annual surface area (identified as permanent or seasonal water) from 1995 to 2015, paired with annual basin-level temperature, precipitation, and population values. With a focus on lake systems, the GLCP can be used to investigate water abundance of the larger hydrologic landscape, as lakes temporally integrate surface and subsurface flows^11,12^. As a result of its interoperability, reproducibility, and extensive coverage, the GLCP is a highly flexible dataset capable of addressing a suite of research questions pertaining to intensive study both within and across specific systems. Additionally, the inclusion of co-located climate and human data as potential drivers of water surface area change makes the GLCP well suited to extensive, multi-scale analysis of heterogeneous environments globally and over decades.

## Methods

### Data sources

#### Lake locations and boundaries

For the locations of lakes, we used the HydroLAKES database version 1.0^13^ (https://www.hydrosheds.org/page/hydrolakes), which incorporates multiple lake datasets (e.g., Shuttle Radar Topology Mission, Water Body Data, Global Lakes and Wetlands Database) and includes 1,427,688 lakes of at least 10 hectares in surface area. The majority of HydroLAKES lakes are defined as uncontrolled lakes (99.5%), with the remainder identified as reservoirs (0.47%) and controlled lakes (0.03%). HydroLAKES, which is available in the form of shapefiles, includes an extensive number of attributes for lake polygons including: lake surface area (polygon area), elevation, shoreline development, total volume, average depth, residence time, latitude and longitude of pour point, lake type, and others. The HydroLAKES v1.0 identifier (“Hylak_id”) is retained in the GLCP to facilitate future work making use of other attributes in the HydroLAKES data, which are not included in the GLCP. Additionally, the GLCP also contains the latitude (“centr_lat”) and longitude (“centr_lon”) of each lake’s centroid, which was calculated within ArcGIS version 3.1^14^.

Hereafter, HydroLAKES lake polygons are referred to as “lakes.”

#### Basins

Because lakes are products of the landscapes in which they reside, we calculated climate and population values relative to each lake’s basin. To identify basin boundaries, we used the HydroBASINS dataset, a basin-level analog to the HydroLAKES dataset. The HydroBASINS version 1.c format 1 database^15^ includes 3,786,218 unique basins and is derived from the HydroSHEDS database^16^, which uses 15 arc-second resolution data to identify river basins, watersheds, and sub-basins globally. In HydroBASINS, basins are identified using the Pfafstetter coding system, with Level 1 as the highest level (i.e., continent level) and Level 12 as the smallest available sub-basin. Table 1 details the number and median size of basins within each Pfafstetter level for basins used within the GLCP. We retain the original HydroBASINS version 1.c identifier (“HYBAS_ID”) for each basin in the GLCP, for ease of future integration with existing HydroBASINS attributes, such as distance from basin outlet to next downstream sink and indicators of endorheic basins.

**Table 1:** Number of HydroLAKES polygons matched with HydroBASINS polygons by Pfafstetter level.

HydroBASIN Pfafstetter level	Number of lakes	Percent of valid lakes	Median basin size (km^2^)
2	2595	0.18%	2198593
3	6650	0.46%	548263
4	8912	0.62%	225882
5	15948	1.12%	58705
6	23262	1.63%	16967
7	41001	2.88%	5042
8	55107	3.87%	1632
9	55061	3.87%	518
10	9747	0.69%	353
11	196	0.01%	333
12	1204020	84.64%	156

Hereafter, HydroBASINS polygons are referred to as “basins.”

#### Surface water extent

For changes in lake surface water area over time, we used the Joint Research Centre (JRC) Global Surface Water Dataset described in Pekel *et al*.^4^, which used LANDSAT imagery (30 meter resolution) from March 1984 through October 2015 to identify changes in surface water area for lakes, rivers, streams, and wetlands. The data are hosted by the European Commission JRC and are formally referred to as the Global Surface Water Dataset. Hereafter, we use the abbreviation “JRC” to refer to this dataset.

The JRC data subsetted for Yearly Water Classification History v1.0 (1984–2015) are publicly available through Google Earth Engine (GEE) as annually aggregated raster images. Each image contains a “waterClass” band with the following values: 0 = no observations, 1 = not water, 2 = seasonal water (defined as water that is present for at least one month but not an entire year), 3 = permanent water (defined as water that is present for all twelve months). For more detailed information on the complete LANDSAT processing workflow used to create the JRC, Pekel *et al*.^4^ provides a methodology of how waterClasses were assigned based on raw LANDSAT data.

While the JRC dataset is the most extensive global surface water dataset available to date, it is limited by the LANDSAT data from which it is derived. Even though LANDSAT coverage began in 1984, portions of northeastern Siberia (Kolyma region and Central Siberian plateau) as well as central Greenland were not included in totality until 1999.

Additionally, the JRC is limited by the water identification algorithm used by Pekel *et al*.^4^, which divided pixels into water, land, or non-valid observations, where non-valid observations may include snow and ice. This system therefore does not classify permanently frozen lakes as water. Seasonally frozen lakes, however, would be coded as entirely seasonal water.

#### Climate data

We used the Modern-Era Retrospective analysis for Research and Applications, Version 2 (MERRA-2)^17^ as the source for climate data. Both precipitation and temperature datasets^18^ were hourly aggregates with original spatial resolution of 0.5 × 0.625 decimal degrees. From these broader datasets, we extracted the variables PRECTOTCORRLAND (total precipitation land; bias corrected; in kg m^−2^ s^−1^, or volumetrically, mm s^−1^) for precipitation and T2MMEAN (2-meter air temperature in K) for temperature. These subsets were exported from NASA Goddard Earth Sciences (GES) and Data Information Services Center (DISC) in a netCDF format for local analysis.

#### Population estimates

We used the Gridded Population of the World (GPW) version 3^19^ for 1995 and GPW version 4^20^ un-adjusted population count data for 2000, 2005, 2010, and 2015 population estimates. Resolution for the GPW version 3 is 2.5 arc-minutes and is available for download from NASA’s Socioeconomic Data and Applications Center (SEDAC). Resolution for GPW version 4 is 30 arc-seconds and is currently hosted on Google Earth Engine. Detailed methodology for the development of these datasets is available in Doxsey-Whitfield *et al*.^21^.

### Data harmonization process

As this project involved harmonizing multiple global datasets at different resolutions, our workflow required multiple steps, each of which resulted in a cleaned data subset. Here we detail the steps taken to integrate the HydroLAKES, HydroBASINS, JRC, climate, and human population datasets described above.

#### Step #1: Calculate lake surface area

Lake surface area for each lake from 1995 to 2015 was calculated using Google Earth Engine^22^. Lake polygons were uploaded and imported into Earth Engine as shapefiles. These lake polygons, which represent typical shape and area for individual lakes, were buffered by a specified distance to allow water area calculations to account for increases in lake area beyond the HydroLAKES polygon borders as specified in HydroLAKES. Buffered lake polygons were then used as boundaries within which to summarize pixels from annual JRC data for each waterClass category (i.e., no data, not water, seasonal water, or permanent water). We calculated “total water” as the sum of seasonal and permanent water pixels. Resulting area values were exported in a .csv format to Google Drive, then downloaded for local analysis using the R statistical environment^23^. Commented Google Earth Engine code for lake area calculations (“jrc_water_class_sum.txt”), the R script for formatting Google Earth Engine output (“01_import_format_JRC.R”)^24–26^, and associated input data are available in the Environmental Data Initiative (EDI) GLCP repository^27^ within the entities “glcp.tar.gz” as well as “glcp_scripts.tar.gz".

To evaluate how lake waterClass areas fluctuated with various buffer sizes, we calculated lake waterClass areas for 1995, 2000, 2005, 2010, and 2015 with 30 m, 60 m, 90 m, and 120 m buffers. Preliminary tests indicated smaller buffers were insufficient to capture large area increases, while larger buffers increased risk of overlapping neighboring lakes (especially in dense lake areas), smaller ponds, or input/output rivers and erroneously increasing lake area totals. Our analysis of waterClass areas between buffer sizes and years indicated 90 m as the most appropriate distance. Additional details are provided in the “Technical Validation” section.

We identified a minority of lakes that were unable to be included in the final data product. One lake in North America was identified as having a broken geometry (Hylak_id = 109424), making it incompatible with Earth Engine-based analyses. Rather than attempt to repair the lake shapefile boundaries and potentially change the size and shape, we chose not to include this lake. Additionally, a small number of lakes were identified to be outside the range of reliable LANDSAT data. The available JRC data has a maximum extent of 80°N and Pekel *et al*.^4^ note that LANDSAT images above 78°N are sparse, partially due to the short LANDSAT observation season in high northern latitudes. As such, we limited further processing to lakes whose entire extent is below 78°N, which excluded 3,220 lakes (0.23% of original 1.4 million lakes).

Given the potential for lakes in this area to have inaccurate area measurements prior to 1999, we calculated ratios of “no data” pixel areas to “not water”, “seasonal water”, “permanent water”, and “total water” pixel areas. These ratios will enable future users to set desired thresholds of “no data” coverage that are specific to their research questions. These ratios are provided within a secondary .csv file (“JRC_all_no_data_proportions_yearly_95thru15.csv”) and can be merged efficiently with the full GLCP using the provided R script (“combine_data_availability_metrics_with_glcp.R”).

#### Step #2: Match lakes with basins

Because HydroBASINS is derived from river networks, rather than lake pour points, the HydroLAKES and HydroBASINS data do not come with a pre-existing 1:1 matching scheme for lake and basin. To match lakes with their equivalent basins, we performed spatial joins for the lake shapefiles and basin shapefiles to identify the smallest basin that enclosed a lake in its entirety. With this basin matching scheme, there is potential for some lakes to be assigned to basins which are larger than their actual basin. As a result, future users are encouraged to compare GLCP-associated basin area to a lake’s known basin area, if those data exist. Comparing HydroBASINS river basins to known lake basins would enable researchers to determine if differences in basin assignment are meaningful of their specific research question. All lakes that fell within a Pfafstetter Level 12 basin (85% of lakes, Table 1) were tagged with the Level 12 basin identifier, because no smaller sub-basins were available. The highest level Pfafstetter basin used was Level 2 (Level 1 being near continent-level), which was sufficient to capture the watersheds of very large lakes, such as the Laurentian Great Lakes and the Caspian Sea. Of the original 3,786,218 HydroBASINS basins, 232,827 were paired with lakes (6.15%). This basin matching procedure was performed within Google Earth Engine (“hylak_hybasin_matching.txt”) and outputs were formatted locally using R (“07_lake_basin_matching.R”)^24,25^.

Using this lake/basin matching procedure, 1,949 lakes (0.14% of the original 1.4 million HydroLAKES lakes) were unable to be properly associated with a basin. Manual investigation indicated that these lakes were either located on islands (645 lakes, 0.05% of the original HydroLAKES) or would be associated with only a Level 1 basin (1,304 lakes, 0.09% of the original HydroLAKES). Lakes located on islands are excluded from the GLCP because their natural basins are not included in the continental basin schema that HydroBASINs employs. Similarly, the 1,304 lakes associated with Level 1 basins were consistently located on the boundary between neighboring basins and therefore never completely enclosed in a single basin. This peculiarity is largely because HydroBASINS is constructed for river networks, as opposed to lakes. Because it is unrealistic for these 1,304 lakes (average total area: 2.39 km^2^) to be influenced by near continental-scale climate and human population forcings, we excluded these lakes from further processing.

#### Step #3: Calculate basin-level precipitation and temperature estimates

Once basins were associated with lakes, basin-level climate values were calculated. Within the R environment, precipitation values from MERRA-2 were converted to annually accumulated precipitation by aggregating hourly data for each gridcell for each year^28,29^. We also derived the average monthly volume of precipitation for each gridcell for each year (1995–2015) by taking the mean of each year’s total monthly precipitation volumes (“summing_hourly_data_precip_mm.R”)^30–33^. Temperature values were similarly used to derive an average annual temperature for each year (“summing_hourly_data_temp_K.R”)^30–34^. The resulting yearly data were saved as rasters. Yearly total precipitation, average monthly total precipitation, and temperature rasters (1995–2015) were then resampled at 1/10^th^ cell size through a bilinear interpolation resampling. The original rectangular grids were converted to squares, with spatial resolution of 0.05 × 0.05 decimal degrees. Because MERRA-2 gridcells were originally sized at 0.5 × 0.625 decimal degree resolution, the initial conversion from a netCDF to a raster format induced extra space (e.g., 90.25°N in raster). As such, resampled rasters were clipped to 90°N/S and 180°W/E and converted to geotiff format for upload to Google Earth Engine (“manipulate_climate_rasters.R”)^30–33^.

For each basin associated with a lake, basin-level average and total precipitation, as well as average temperature, were calculated for each year of interest in Earth Engine. The process was similar to the one described for lake polygons and JRC data, whereby the basins were used as boundaries from which to extract and aggregate pixels. Results were exported as .csv files to Google Drive, then downloaded for local analysis using R. R scripts for data aggregation of climate variables (“04_post_gee_processing_temp.R”, “05_post_gee_processing_precip_sum.R”, “06_post_gee_processing_precip_average.R”)^24,25,35^ are available on the EDI GLCP repository^27^ within the entity “glcp.tar.gz”.

This process resulted in 10 matched basins (of the original 232,837 matched basins; 0.004%), which were associated with 19 lakes, with missing values for climate variables. These 10 basins ranged in size from 1.1 to 181.7 km^2^ with a median area of 76.5 km^2^. These basins and lakes were removed from the dataset. Manual assessment showed that these basins were located at higher northern latitudes in the United States and the Russian Federation. We note that other temperature and precipitation datasets are available; subsequent analyses can incorporate alternative climate data sources to match with these basins through the scripts and workflow provided in the EDI entity “glcp.tar.gz”.

Future users should also note that while HydroBASINS provided a boundary to calculate climate variables for each lake, these calculations may be overestimates, as many lakes’ actual basins may be smaller than their associated river basin. However, as with the addition of new climate variables, subsequent analyses can also incorporate different basin schemes as lake basin shapefiles become available.

#### Step #4: Calculate basin-level population estimates

While other data sources in this project are annual, the global population data we used, which was the current best available at the global scale, was for 5-year increments (1995, 2000, 2005, 2010, 2015). Rather than interpolate the intervening years’ values, we chose to leave these blank so that future researchers can personalize statistical methodology to best address these data gaps in context of a specific question. Aside from blank values, numerous basins have population estimates as decimal values. Rather than truncate these values which were produced through the aggregation process within Google Earth Engine, we retain these so that future users may decide how they wish to round or otherwise interpret these values in the context of their particular research question.

For each 5-year increment, human population calculations were performed with a technique similar to the climate data aggregations. GPWv3 (1995 data) was converted to a geotiff and imported into Earth Engine. GPWv4 (2000, 2005, 2010, and 2015) rasters were available through the Earth Engine interface. Basin-level population totals were calculated from GPWv3 and GPWv4 data with basin polygons as spatial boundaries. Results were exported as .csv files to Google Drive and then downloaded for local analysis within the R environment. R scripts for data aggregation of population counts (“02_load_shp_GPWv3.R”, “03_load_shp_GPWv4.R”)^24,25,35^ are available on the EDI GLCP repository^27^ within the entity “glcp.tar.gz”.

Like climate variables, population calculations have the potential to be overestimated, as many lakes’ actual basins may be smaller than their associated river basin. Additionally, future users should be cognizant of heterogeneous human populations within the basin, which could skew analyses of relationships between human population and lake area. As is currently calculated, population estimates are at the basin scale, whereas certain research questions may be more focused at population estimates within a particular distance from the lake (e.g., 500 m). However, subsequent analyses can also incorporate different basin schemes as lake basin shapefiles become available.

#### Step #5: Merge lake- and basin-level data

Lake- and basin-level output were merged within the R environment. The R script for GLCP production (“08_cleaning_glcp_production.R“)^24–26^ is available in the EDI GLCP repository within the entity “glcp.tar.gz”.

Synopsis of lake exclusions during data harmonization:

1. One lake in North America contained broken geometry (Hylak_id = 109424) and was incompatible with Earth Engine-based analyses (99.99% of original lakes were retained)
2. 3,220 lakes extended beyond 78°N, maximum reliable extent of JRC data (99.77% of original lakes were retained)
3. 1,949 lakes were unable to be associated with a level 02–12 basins (99.64% of original lakes were retained)
4. 19 lakes (from 10 unique basins) were spatially outside range of temperature or precipitation MERRA-2 data (99.64% of original lakes were retained)

**Fig. 1:**
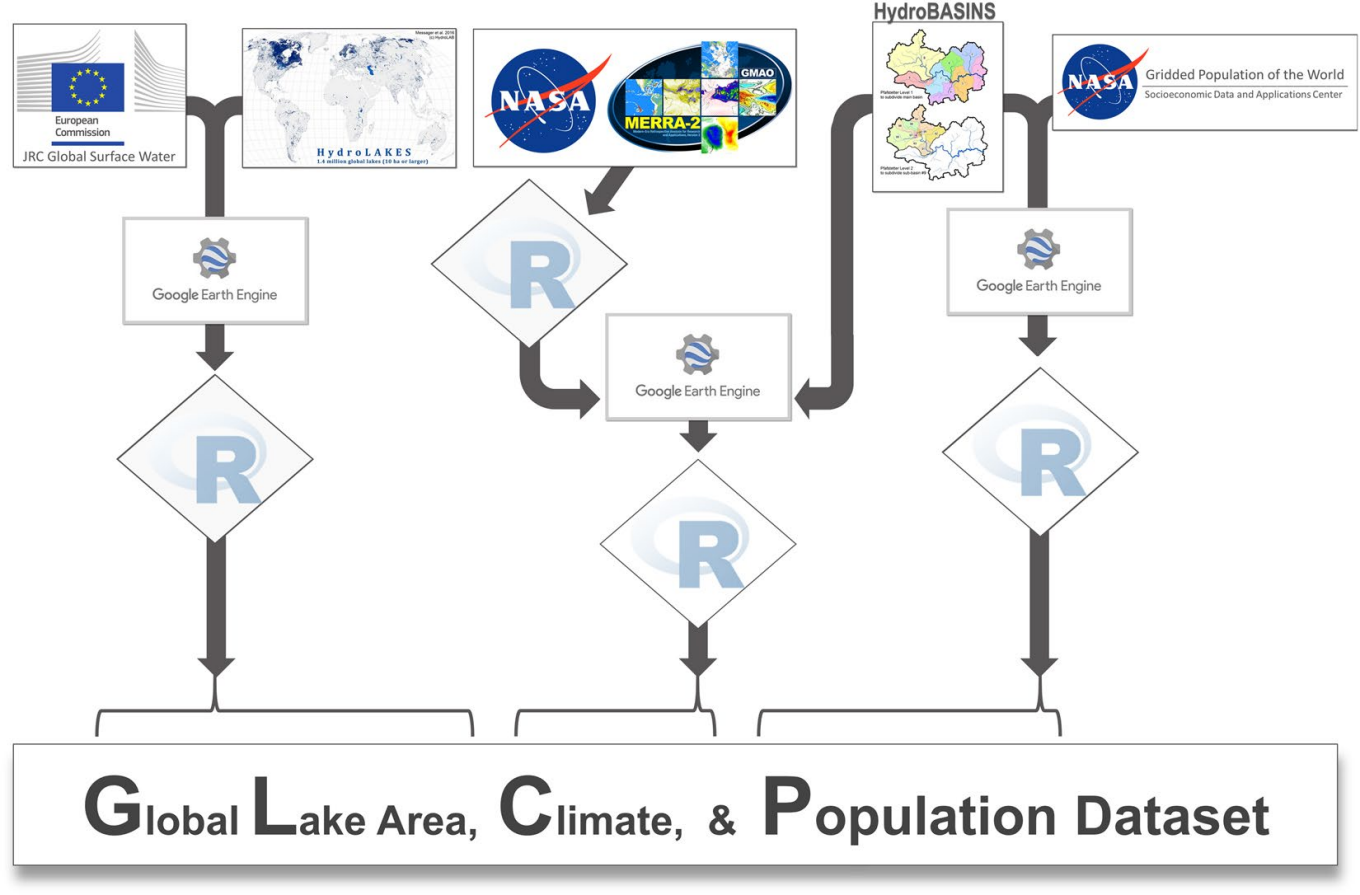
Conceptual diagram of data harmonization process. JRC Water area was calculated in Google Earth Engine (GEE), and then aggregated locally within the R environment. Similarly, basin population was calculated on GEE, and then aggregated locally within the R environment. Climate data from MERRA-2, however, was first aggregated from hourly to monthly and annual values, then resampled for 1/10th of their original resolutions with a subsequent and raster clipping was performed within the R environment. We then imported the geotiff outputs to GEE to calculate basin climate variables. GEE output was then downloaded locally and aggregated within the R environment.

A conceptual diagram of the harmonization process is provided in Fig. 1.

The final GLCP dataset contains 1,422,499 lakes. These lakes were used to generate all dataset summary statistics reported below.

## Data Records

Final data are provided in the form of one .csv file (“glcp.csv”). The GLCP can be found in the Environmental Data Initiative repository^27^. The repository also contains calculated values for quality control for a given lake and year that can also be downloaded in a separate .csv file (“JRC_all_no_data_proportions_yearly_95thru15.csv”). Future users may wish to combine these data quality metrics with the GLCP, and then set thresholds for their own analyses. While numerous potential thresholds exist, the authors encourage reporting any data metric thresholds used in GLCP applications, so as to maintain future reproducibility of GLCP analyses.

The GLCP (“glcp.csv”) has a tabular set-up as described below:

*year*

Year, spans 1995–2015. Note that for the purposes of these data 2015 ends on October 31, consistent with the JRC data.

*Hylak_id*

HydroLAKES unique identifier of lake. Preserved from HydroLAKES input data to enable future merge with HydroLAKES attributes.

*centr_lat*

Lake centroid latitude.

*centr_lon*

Lake centroid longitude.

*continent*

Continent on which lake is located (from HydroLAKES dataset).

*country*

Country in which lake is located (from HydroLAKES dataset).

*bsn_lvl*

Pfafstetter level of basin associated with lake.

*HYBAS_ID*

HydroBASINS unique identifier of basin associated with lake. Preserved from HydroBASINS input data to enable future merging with HydroBASINS attributes.

*mean_monthly_precip_mm*

Mean monthly basin-level precipitation, in mm.

*total_precip_mm*

Total annually accumulated basin-level precipitation, in mm.

*mean_annual_temp_k*

Mean annual basin-level temperature, in Kelvin.

*pop_sum*

Total basin-level human population. Note that this column only has valid values for 1995, 2000, 2005, 2010, and 2015. NA values are listed for all other years.

*seasonal_km* ^*2*^

Water area, in square kilometers, of “seasonal” water, as defined by Pekel *et al*.^4^.

*permanent_km* ^*2*^

Water area, in square kilometers, of “permanent” water, as defined by Pekel *et al*.^4^.

*total_km* ^*2*^

Calculated “total” water, in square kilometers, as the sum of seasonal and permanent water.

### Quality control variables

Additional waterClasses and calculated ratios between waterClasses for each lake and year are contained in a separate .csv file (“JRC_all_no_data_proportions_yearly_95thru15.csv”). Data availability metrics can be joined with the GLCP using the R script “combine_data_availability_metrics_with_glcp.R”.

*no_data_km* ^*2*^

Area of 90 m buffered lake, in square kilometers, of no data, as defined by Pekel *et al*.^4^.

*not_water_km* ^*2*^

Area of 90 m buffered lake, in square kilometers, of not water, as defined by Pekel *et al*.^4^.

*no_data_to_not_water*

Calculated ratio of no_data_km^2^ derived area to not_water_km^2^ derived area.

*no_data_to_seasonal*

Calculated ratio of no_data_km^2^ derived area to seasonal_km^2^ derived area.

*no_data_to_permanent*

Calculated ratio of no_data_km^2^ derived area to permanent_km^2^ derived area.

*no_data_to_total*

Calculated ratio of no_data_km^2^ derived area to total_km^2^ calculated area.

## Technical Validation

### Lake buffer size comparison

As mentioned above, we tested multiple lake buffer sizes, to capture fluctuations in water area that extend beyond the static border of the original HydroLAKES shapefiles. We calculated waterClass areas for lakes with 30 m, 60 m, 90 m, and 120 m buffers at five-year intervals between 1995 and 2015. We chose to evaluate buffer sizes using a subsample of years in order to balance extensive temporal coverage with reduced computing time. The five-year increments captured a range of LANDSAT coverage completeness, where 1995 and 2000 represented years with more spotty coverage and 2005, 2010, and 2015 represented years with more consistent coverage. Ideally, water area values would increase, then plateau with increasing buffer distance, indicating that the buffer had captured all available water area changes of the lake. A continual increase in water area with buffer distance could indicate either very large increases in water area beyond typical lake boundaries or capture of non-target lake water, such as nearby ponds, rivers, or neighboring lakes.

We approached the issue of appropriate buffer distance in two ways: (1) comparing observed total water area to HydroLAKES expected lake area and (2) assessing proportional water increases with increasing buffer sizes across lakes. The first approach also served as validation that our methods were returning values on the same scale as expected.

To validate GLCP-calculated lake areas, we compared the observed total water area of all 1,422,499 valid lakes and reservoirs to their expected areas as provided in the HydroLAKES dataset, with 30 m, 60 m, 90 m, and 120 m buffers (Table 2). Across all buffers and years included, the ratio of observed to expected values had an approximate mean of 0.9 (Table 2). The median and mean for 1995 were smaller than 0.9, for all buffers, which was expected because of the higher number of zero values in 1995 due to the gaps in LANDSAT coverage.

**Table 2:** Ratios of JRC-derived total water area to water area as reported in HydroLAKES for five year intervals (N = 1,422,499 lakes).

Buffer (m)	Year	Min.	1st Qu.	Median	Mean	3rd Qu.	Max.
**30**	**1995**	0.000	0.414	0.874	0.690	0.968	2.106
	**2000**	0.000	0.795	0.906	0.863	0.991	2.205
	**2005**	0.000	0.813	0.919	0.870	0.997	2.181
	**2010**	0.000	0.803	0.915	0.864	0.997	2.301
	**2015**	0.000	0.818	0.921	0.871	0.997	2.515
**60**	**1995**	0.000	0.424	0.879	0.703	0.975	3.185
	**2000**	0.000	0.805	0.917	0.891	1.011	3.481
	**2005**	0.000	0.824	0.930	0.898	1.017	3.367
	**2010**	0.000	0.814	0.925	0.892	1.017	3.769
	**2015**	0.000	0.829	0.931	0.900	1.017	4.095
**90**	**1995**	0.000	0.432	0.883	0.714	0.981	4.208
	**2000**	0.000	0.812	0.924	0.913	1.025	4.769
	**2005**	0.000	0.831	0.937	0.921	1.032	4.579
	**2010**	0.000	0.821	0.932	0.915	1.031	5.287
	**2015**	0.000	0.835	0.938	0.924	1.032	5.667
**120**	**1995**	0.000	0.440	0.889	0.726	0.989	5.349
	**2000**	0.000	0.818	0.931	0.935	1.041	6.240
	**2005**	0.000	0.839	0.945	0.944	1.048	5.793
	**2010**	0.000	0.829	0.940	0.938	1.047	6.891
	**2015**	0.000	0.843	0.946	0.948	1.049	7.213

Values greater than one indicate observed values greater than expected, and values smaller than one indicate smaller than expected values.

In selecting the optimal buffer size, we wanted to minimize the proportion of lake area increases due to increased buffer distance capturing nearby waterbodies, as opposed to true increase in lake size. We focused our analysis on the proportion of lakes that increase >1% in total water area as buffer distance increased. This percentage was lowest between 60 m and 90 m (Table 3). As the buffer was extended to 120 m, the proportion of lakes displaying >1% increase also increased. While this proportional increase may be valid in some cases, we broadly interpret this change to represent capture of nearby waterbodies as the buffer increases from 90 m to 120 m. Lakes increasing greater than 1%, 5%, and 10% for all calculated years and buffer sizes accounted for 13.2%, 5.6%, and 1.9% of all GLCP lakes, respectively. These results suggest that if a GLCP lake was influenced by neighboring waterbodies, it would at most account for a > 1% increase in 13.2% of GLCP lakes, >5% increase for 5.6% of GLCP lakes, and a > 10% increase for 1.9% of GLCP lakes. Summary statistics for lakes increasing at least 1% are documented in Table S1.

**Table 3:** Buffer comparisons for five year intervals with percent of lakes (N = 1,422,499) <1% increase, 1–5% increase, 5–10% increase, and >10% increase in total water surface area.

Buffer:Buffer	Size Change	1995	2000	2005	2010	2015
**60 m:30 m**	**<1% increase**	55.00%	59.62%	58.32%	58.98%	58.93%
	**>1% increase**	15.64%	20.16%	20.81%	20.18%	20.22%
	**>5% increase**	4.49%	8.62%	8.78%	8.58%	8.49%
	**>10% increase**	3.65%	9.86%	10.14%	10.23%	10.44%
	**Decrease**	0.01%	0.01%	0.01%	0.01%	0.01%
	**Zero water area**	21.21%	1.74%	1.93%	2.02%	1.91%
**90 m:60 m**	**<1% increase**	58.01%	64.68%	63.62%	64.04%	63.86%
	**>1% increase**	14.64%	19.65%	20.10%	19.53%	19.49%
	**>5% increase**	3.72%	7.66%	7.85%	7.83%	7.83%
	**>10% increase**	2.44%	6.29%	6.52%	6.61%	6.95%
	**Decrease**	0.03%	0.03%	0.03%	0.03%	0.03%
	**Zero water area**	21.16%	1.70%	1.88%	1.96%	1.84%
**120 m:90 m**	**<1% increase**	53.23%	61.18%	59.89%	60.30%	59.90%
	**>1% increase**	18.11%	23.72%	24.37%	23.86%	23.92%
	**>5% increase**	4.91%	8.53%	8.78%	8.72%	8.91%
	**>10% increase**	2.61%	4.88%	5.10%	5.18%	5.46%
	**Decrease**	0.02%	0.02%	0.02%	0.02%	0.02%
	**Zero water area**	21.12%	1.65%	1.83%	1.91%	1.79%

**Fig. 2:**
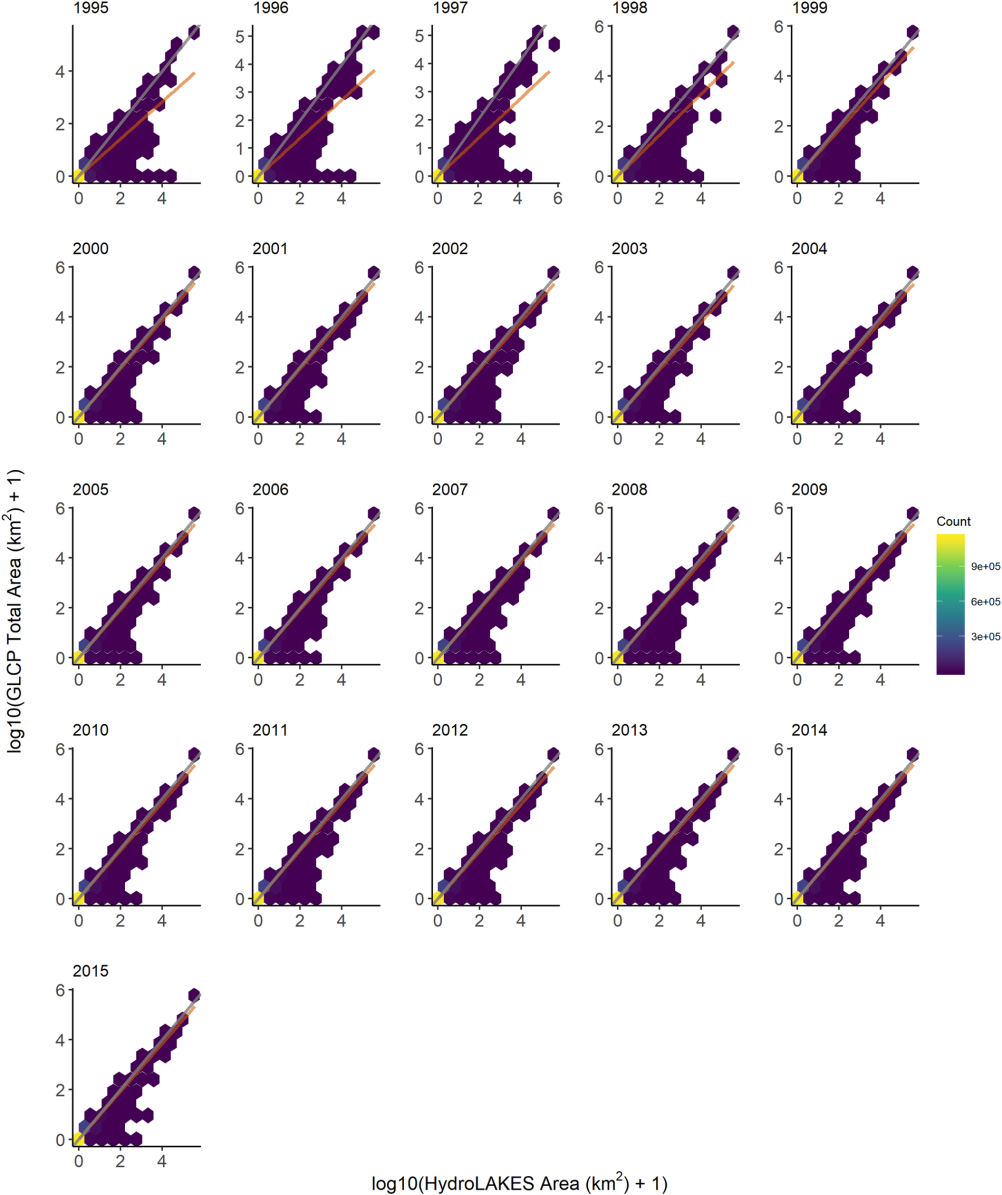
Comparison of 90 m buffer total water areas from JRC to reported lake areas in HydroLAKES. The grey diagonal line is the 1:1 line, where a value on the 1:1 grey line implies no difference between data sources. Color of hexbin indicates the number of lakes contained within a bin. The orange line is a linear regression for all points. The orange regression line tends to deviate more markedly for comparisons among the mid- to late-1990s. This is likely due to sparse LANDSAT coverage during the mid-to-late 1990s. Starting in 1999, though, the orange and grey lines are nearly identical, which would be expected as LANDSAT coverage in 1999 and 2000 incorporated more complete global coverage, particularly in Siberia and Greenland. Scales have been log-transformed in order to show a more even spread of data points, as most lakes are less than 10 km^2^.

As a result of proportional increases from 90 m to 120 m, we determined that a 90 m buffer was the best option for minimizing under- and over-estimation of total water area. Interannual comparisons of HydroLAKES areas to GLCP-calculated total areas also demonstrated relatively consistent values for all lakes across all years (Fig. 2; Table S2). Likewise, the same pattern was observed when comparing interannual GLCP-calculated water areas (Table S3). While 1995–1998 interannual comparisons deviated more markedly from the 1:1 line (Fig. 2), this is largely an artifact of LANDSAT coverage missing from 1996–1998^4^.

### Lakes with zero total area values for all years

Pekel *et al*.^4^ report validation results for the JRC dataset, including errors by sensor type and seasonality class. Commission accuracy ranges from ~98–99% while omission accuracy varies more widely, from ~74–99%, with lowest accuracy for seasonal water pixels. Of the 1,422,499 lakes in the GLCP, only 12,534 lakes had a zero total water area value for all time points (i.e., water never identified within lake borders). Therefore, we have approximately 99% accuracy in terms of detecting water within a lake for the global lakes considered in the GLCP.

Assuming 99% accuracy in water detection, lakes with zero values for total water area represent a suite of possibilities. First, zeroes could represent real instances of zero water surface area (i.e., lake has dried up). Second, zeros could represent real missing (NA) values from incomplete LANDSAT coverage for certain locations and years. Third, zero area values could represent a mis-identified HydroLAKES polygon (i.e., not an actual lake in that location)^36^. Fourth, zero values could be due to permanently frozen lakes, which the JRC algorithm does not identify as “water”. Temporal location of zero area lakes can guide interpretation of zero values. For instance, a zero value for 2005, but non-zero values for all other time points of interest, potentially indicates a “true” zero (i.e., a lake drying and refilling or a lake being frozen one year and thawing the next). Likewise, a lake with non-zero values for 1995–2005, with zero values subsequently, suggests a scenario of a lake drying and remaining dry. Conversely, zeros for 1995 only or 2015 only are more challenging to interpret: as end points of our temporal range, it is difficult to determine whether these zero values are true zeros or NA values. Temporal location of zero values is of less assistance in cases where lakes may be permanently frozen and therefore pixels were categorized as not water.

### Climate comparison

While the JRC and GPW datasets represent the best available global datasets to date for water extent and population for our time range, there are multiple sources possible for global, long-term climate data. We selected MERRA-2 for GLCP climate data because MERRA2 had the finest spatial and temporal resolutions in comparison to similar global climate datasets. Additionally, along with including various temperature and precipitation variables, MERRA-2 includes a wide variety of other climate variables should future users wish to expand this workflow to investigate other possible correlates. To test that our workflow maintained the integrity the MERRA-2 data, as well as check for general scale of differences in results compared to alternative climate data sources, we compared MERRA2-derived climate data to equivalent basin-scale results derived from the University of Delaware Air Temperature and Precipitation V5.01 (UDATP) dataset^37^. The UDATP is a global gridded climate dataset with 0.05 × 0.05 decimal degree resolution of monthly average air temperature and accumulated precipitation.

Both MERRA-2 and UDATP mean annual temperature and mean monthly precipitation were broadly consistent throughout the 21 years. For temperature, greater incongruences between UDATP and MERRA2 were observed at colder temperatures, whereas warmer temperatures tended to be more consistent between data sources (Fig. S1). For precipitation, we observed greater deviations from the 1:1 line, although trend lines were similar to a 1:1 trend (Fig. S1). The discrepancies between MERRA-2 and UDATP are consistent with previous observations that these discrepancies are likely due to how UDATP and MERRA-2 incorporate observation-based corrections^38^.

Given the potential changes in interpretation of lake area change in the context of temperature and precipitation variables based on which climate dataset is used, we recommend future users interested in understanding how climate may correlate with or predict lake area should compare various climate datasets.

### Manual quality control

To ensure integrity of data through each level of data harmonization, we sampled 250 lakes and manually evaluated their year-to-year percent differences (e.g., $(\frac{total\_k{m}_{1995}^{2}-total\_k{m}_{1996}^{2}}{\frac{total\_k{m}_{1995}^{2}+total\_k{m}_{1996}^{2}}{2}})\ast 100$) in area and climate variables through visual inspection of individual lake values (Table 4). A stratified random sample of 250 lakes was collected via the following process: We used continent-level shapefiles to spatially subset a dataset of HydroLAKES centroids into separate datasets for each continent. A random sample of HydroLAKES Hylak_ids was then taken from each continent’s dataset based on the proportion of lakes in the full HydroLAKES dataset originating from that continent. In some cases we sampled an additional lake to ensure that a full sample of 250 Hylak_ids was achieved when rounding would otherwise have led to a sample size smaller than 250. Variables were *a priori* hypothesized to have an overall low degree of interannual variation by assuming that most lakes do not undergo large interannual changes in area and climate. In instances of high variation, this process would identify specific outliers that could be explained by local anomalies (e.g., extensive drought, ENSO). Of the 250 lakes and basins sampled, interannual percent differences for climate variables (i.e., temperature, total annual precipitation, and mean monthly precipitation) were smallest (Table 4). Water area interannual percent differences were smallest for permanent and total water, whereas seasonal water area had a comparably larger range of interannual percent differences (Table 4). Population interannual percent differences were highest among all variables (Table 4), which is likely due to the coarser temporal resolution of the population data. Percent difference values ranging from −200% to +200% indicates that variables went from a value of zero to a non-zero value. Because values consistently remained within a fairly constant range of year-to-year values, this comparison suggests that data integrity did not substantially degrade during the harmonization process.

**Table 4:** Summary table (minimum, 1^st^ quartile, median, mean, 3^rd^ quartile, and maximum) of interannual percent difference for 250 spatially stratified lakes used for manual QA/QC.

Variable	Min.	1st Qu.	Median	Mean	3rd Qu.	Max.
total_precip_mm	−98.64	−13.38	−0.62	−0.41	11.85	95.77
mean_monthly_precip_mm	−98.64	−12.86	−0.24	0.02	12.16	95.77
mean_annual_temp_k	−1.74	−0.34	0.02	0.02	0.36	1.78
pop_sum	−200.00	−42.27	11.88	−42.08	2.17	198.98
seasonal_km^2^	−200.00	−31.89	0.88	3.34	37.63	200.00
permanent_km^2^	−200.00	−2.58	0.00	1.58	2.65	200.00
total_km^2^	−200.00	−2.54	0.00	2.14	2.83	200.00

The range of percent differences was bounded between −200 and +200 percent, implying a year-to-year change in value from zero to non-zero.

To ensure integrity of the data across countries, we randomly sampled 60 countries from those represented within the GLCP (33.8%), and compared ranges of values for each variable with reported values from various online resources (e.g., World Bank, United Nations). This process confirmed that observed values and ranges for each calculated lake area, climate, and human demographic variables were largely consistent with independently reported values.

### Quantifying effects of processor heterogeneity

When recreating the GLCP from its original inputs, we noted slight (e.g., 1e-12) differences between script runs. Because production of the GLCP required a heterogeneous computational architecture (in our case, Google Earth Engine), floating-point values may vary depending on the make and model of processor used if users create the GLCP de novo^39^. While particular processors in the Google Earth Engine API were likely heterogeneous and not specified, post-GEE processing was completed with a homogeneous Intel Xeon Processor E5-2680 v2 (ivybridge) consisting of 20 cores (2 sockets and 10 cores per socket) and 256 GB memory. To quantify differences in GLCP-derived values as a result of processor heterogeneity, we recreated the GLCP three times and calculated percent difference for each computed value within the R environment (Table S4). Percent differences were very low for each variable (Table S4) and are likely not significant for most future applications. Care, however, should be taken if users recreate the GLCP from its raw inputs, as potential differences in floating decimals may lead to slightly different calculated values. The extent to which these values would influence results or conclusions of future studies will depend on the level of precision required.

## Usage Notes

The GLCP can be applied to a suite of research situations pertaining to lake water quantity, including using water area as a proxy for volume when such data are not available. We highlight two main areas for immediate application.

First, the GLCP can be joined with other global hydrologic and climate datasets to synthesize global changes in water quantity and quality. For example, the GLCP could be merged with measurements of change in subsurface water quantity from GRACE data^3^ to assess how changes in lake water quantity may co-vary with changes in groundwater quantity. Similarly, the GLCP could be merged with lake water quality data from organizations such as the Global Lake Ecological Observatory Network (GLEON)^40^ to address questions of how changes in lake water quantity may co-vary with changes in lake water quality. Beyond incorporating existing lake-related data sources, other climate variables (e.g., wind speed, humidity) could be calculated and incorporated by using the HydroBASINS shapefiles and Google Earth Engine in a similar process to that described above. Aside from adding new variables, the framework used to produce the GLCP is flexible enough to incorporate new lake and basin shapefiles (e.g., GLOWABO^2^). While the GLCP focuses on lakes of at least 10 ha in size, lakes smaller than 10 ha may be necessary for addressing ecological and biogeochemical questions and in evaluating continental- and global-scale carbon cycling^41,42^ as well as spatio-temporal variation in lake ecosystem properties^43^.

Second, the GLCP can be used to create a common georeference for multi-source data to increase research efficiency by allowing users to quickly assimilate disparate data for location-specific studies. Similar to the manner in which the U.S. Environmental Protection Agency’s National Lake Assessment (NLA) has been used to compare individual lake’s water quality to that of the region^44^, the GLCP can be used to: (1) compare how a given lake’s surface area may compare to any other lake’s during any given year and (2) create a time series of lake surface area as well as basin climate and population. In doing so, the GLCP could be used by local natural resource managers by providing long-term lake characteristics as a potential explanation for biological phenomena.

Given its potential for future applications, the GLCP dataset serves not only as a streamlined resource for exploring changes in lake-specific surface area over the past two decades, but also provides future data users a powerful tool to contextualize lake area changes with co-located climate and population data.

## Supplementary information

Supplemental Information

## Data Availability

All Google Earth Engine and R scripts are available from the Environmental Data Initiative GLCP repository^27^ within the entity “glcp.tar.gz”. The entity “glcp.tar.gz” also includes a standardized directory architecture that can be downloaded and run locally if users wish to reproduce the GLCP. If future users would like to access only scripts used to create the GLCP, the EDI GLCP repository also contains the entity “glcp_scripts.tar.gz”, which contains Google Earth Engine and R scripts.
