# Supplementary material for: The global lake area, climate, and population dataset
Source: Sci Data. 2020 Jun 11;7:174. doi: 10.1038/s41597-020-0517-4 (PMC7289843; doi:10.1038/s41597-020-0517-4)
Supplement: Supplementary file 1 — Supplemental Information [file 41597_2020_517_MOESM1_ESM.docx]

Table S1: Summary statistics of lake area, basin climate, and basin human population variables for lakes that increased at least 1% in area for all buffer sizes and years assessed (i.e., 1995, 2000, 2005, 2010, 2015).

| **Variable** | **Min.** | **1st Qu.** | **Median** | **Mean** | **3rd Qu.** | **Max.** |
| --- | --- | --- | --- | --- | --- | --- |
| *total_precip_mm* | 0.88 | 455.92 | 613.25 | 704.47 | 773.66 | 7523.79 |
| *mean_monthly_precip_mm* | 0.08 | 38.20 | 51.32 | 58.94 | 64.66 | 683.98 |
| *mean_annual_temp_k* | 252.88 | 262.30 | 266.60 | 270.91 | 272.77 | 304.01 |
| *pop_sum* | 0 | 7.63 | 66.87 | 1.32E+05 | 420.07 | 5.27E+08 |
| *seasonal_km2* | 0.00 | 0.00 | 0.01 | 0.07 | 0.05 | 4.08 |
| *permanent_km2* | 0.00 | 0.02 | 0.14 | 0.17 | 0.24 | 6.23 |
| *total_km2* | 0.00 | 0.09 | 0.19 | 0.24 | 0.30 | 9.01 |

Table S2: Summary statistics of interannual comparisons for each lake contained in the GLCP with the same lake’s area as defined in HydroLAKES. A value less than one indicates the JRC-derived area as less than the HydroLAKES reported area, whereas a value greater than one indicates the JRC-derived area as greater than the HydroLAKES area. Slightly higher mean deviations are observed in the mid-to-late 1990s in comparison to those after 2000, likely as a result of sparse satellite coverage. Even with these deviations, the data are consistent from year-to-year, giving confidence in interannual consistency for included lakes.

| **Year** | **Min.** | **1st Qu.** | **Median** | **Mean** | **3rd Qu.** | **Max.** |
| --- | --- | --- | --- | --- | --- | --- |
| **1995** | 0.000 | 0.452 | 0.884 | 0.716 | 0.981 | 4.208 |
| **1996** | 0.000 | 0.000 | 0.876 | 0.685 | 0.977 | 5.385 |
| **1997** | 0.000 | 0.007 | 0.863 | 0.676 | 0.969 | 5.508 |
| **1998** | 0.000 | 0.716 | 0.897 | 0.786 | 0.992 | 4.400 |
| **1999** | 0.000 | 0.794 | 0.920 | 0.867 | 1.012 | 5.413 |
| **2000** | 0.000 | 0.813 | 0.924 | 0.915 | 1.026 | 4.769 |
| **2001** | 0.000 | 0.824 | 0.930 | 0.918 | 1.025 | 5.369 |
| **2002** | 0.000 | 0.822 | 0.930 | 0.909 | 1.021 | 4.830 |
| **2003** | 0.000 | 0.800 | 0.916 | 0.881 | 1.003 | 4.408 |
| **2004** | 0.000 | 0.828 | 0.935 | 0.916 | 1.030 | 5.731 |
| **2005** | 0.000 | 0.832 | 0.937 | 0.923 | 1.032 | 4.579 |
| **2006** | 0.000 | 0.835 | 0.939 | 0.926 | 1.034 | 5.714 |
| **2007** | 0.000 | 0.830 | 0.937 | 0.926 | 1.037 | 5.335 |
| **2008** | 0.000 | 0.827 | 0.935 | 0.916 | 1.031 | 5.558 |
| **2009** | 0.000 | 0.834 | 0.940 | 0.929 | 1.040 | 5.722 |
| **2010** | 0.000 | 0.822 | 0.933 | 0.917 | 1.031 | 5.287 |
| **2011** | 0.000 | 0.825 | 0.938 | 0.923 | 1.040 | 5.740 |
| **2012** | 0.000 | 0.760 | 0.892 | 0.868 | 0.996 | 5.665 |
| **2013** | 0.000 | 0.838 | 0.940 | 0.932 | 1.038 | 5.740 |
| **2014** | 0.000 | 0.842 | 0.943 | 0.938 | 1.042 | 5.636 |
| **2015** | 0.000 | 0.836 | 0.939 | 0.926 | 1.032 | 5.667 |

Table S3: Summary statistics of interannual comparisons for each lake area contained within the GLCP. A value less than one indicates the latter year’s area as greater than the former year’s area, whereas a value greater than one indicates the latter year’s area as less than the former year’s area. Slightly higher mean deviations are observed in the mid-to-late 1990s in comparison to those after 2000, suggesting remnants from sparse satellite coverage. Even with sparse satellite coverage, the data are consistent from year-to-year, giving confidence in interannual consistency for included lakes.

| **year:year** | **Min.** | **1st Qu.** | **Median** | **Mean** | **3rd Qu.** | **Max.** |
| --- | --- | --- | --- | --- | --- | --- |
| **1995:1996** | 0.000 | 0.973 | 1.000 | 2.059 | 1.026 | 99236.252 |
| **1996:1997** | 0.000 | 0.977 | 1.003 | 2.109 | 1.034 | 99831.287 |
| **1997:1998** | 0.000 | 0.926 | 0.993 | 1.272 | 1.022 | 55846.619 |
| **1998:1999** | 0.000 | 0.926 | 0.989 | 1.875 | 1.013 | 187756.835 |
| **1999:2000** | 0.000 | 0.957 | 1.005 | 1.163 | 1.046 | 19800.238 |
| **2000:2001** | 0.000 | 0.965 | 0.997 | 1.519 | 1.020 | 40847.822 |
| **2001:2002** | 0.000 | 0.975 | 1.000 | 1.832 | 1.021 | 96708.661 |
| **2002:2003** | 0.000 | 0.985 | 1.003 | 1.972 | 1.044 | 53962.583 |
| **2003:2004** | 0.000 | 0.948 | 0.992 | 1.347 | 1.008 | 26219.437 |
| **2004:2005** | 0.000 | 0.982 | 1.000 | 1.325 | 1.019 | 37402.580 |
| **2005:2006** | 0.000 | 0.980 | 1.000 | 1.280 | 1.015 | 35725.069 |
| **2006:2007** | 0.000 | 0.985 | 1.000 | 1.278 | 1.020 | 52901.691 |
| **2007:2008** | 0.000 | 0.984 | 1.000 | 1.562 | 1.023 | 46028.962 |
| **2008:2009** | 0.000 | 0.975 | 0.998 | 1.187 | 1.014 | 15288.290 |
| **2009:2010** | 0.000 | 0.988 | 1.002 | 1.299 | 1.028 | 16393.431 |
| **2010:2011** | 0.000 | 0.975 | 0.998 | 1.330 | 1.013 | 34720.477 |
| **2011:2012** | 0.000 | 1.005 | 1.035 | 1.621 | 1.101 | 44756.699 |
| **2012:2013** | 0.000 | 0.905 | 0.963 | 1.179 | 0.992 | 13833.978 |
| **2013:2014** | 0.000 | 0.988 | 1.000 | 1.064 | 1.007 | 3376.273 |
| **2014:2015** | 0.000 | 0.993 | 1.000 | 1.257 | 1.015 | 21168.566 |

Table S4: Summary statistics of percent differences in GLCP values when created de novo. Despite being created from the same data and source code, floating decimal values may vary due to heterogeneities in processor architecture.

| **Variable** | **Min.** | **1st Qu.** | **Median** | **Mean** | **3rd Qu.** | **Max.** |
| --- | --- | --- | --- | --- | --- | --- |
| *total_precip_mm* | -2.26E-13 | 0 | 0 | -2.01E-19 | 0 | 0 |
| *mean_monthly_precip_mm* | -2.89E-13 | 0 | 0 | -2.37E-20 | 0 | 0 |
| *mean_annual_temp_k* | -3.53E-13 | 0 | 0 | 3.06E-21 | 0 | 3.94E-13 |
| *pop_sum* | -8.54E-13 | 0 | 0 | 9.04E-19 | 0 | 8.05E-13 |
| *seasonal_km2* | -9.63E-13 | 0 | 0 | -8.31E-20 | 0 | 9.59E-13 |
| *permanent_km2* | -7.76E-13 | 0 | 0 | 5.07E-20 | 0 | 9.62E-13 |
| *total_km2* | -9.88E-13 | 0 | 0 | -1.37E-20 | 0 | 9.57E-13 |


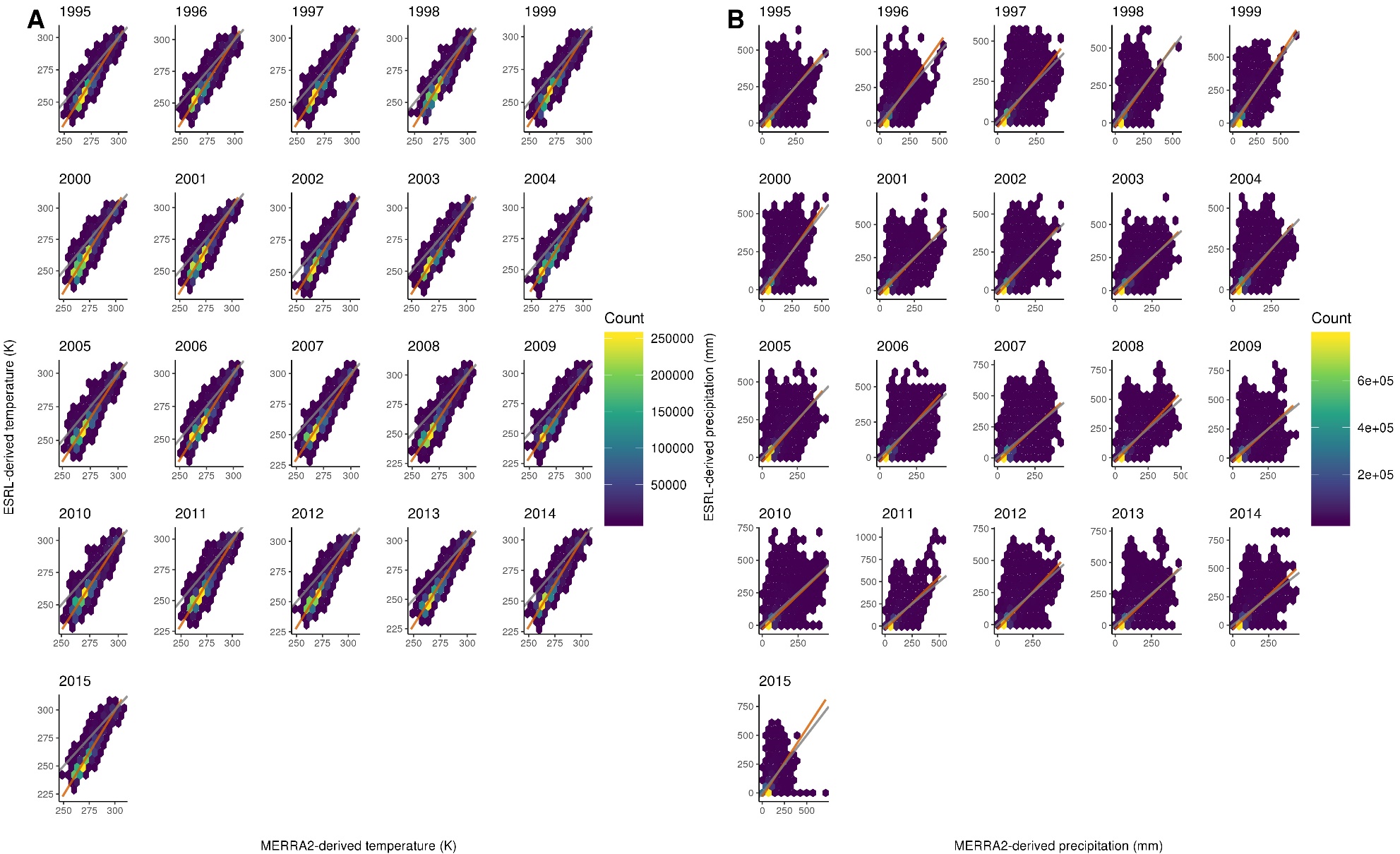


Figure S1: Comparison of basin-level MERRA2-derived climate data with University of Delaware Air Temperature and Precipitation (UDATP)-derived climate data. Each comparison plot contains a grey 1:1 line, an orange linear regression line, and hexbins colored by number of basins. Comparisons for temperature data (A) demonstrate greater congruence between the two datasets at higher temperatures as opposed to lower temperatures. Throughout the time series, discrepancies between UDATP- and MERRA2-derived temperature values remain consistent. Differences between precipitation values (B) vary more than temperature values. Despite precipitation comparisons deviating from the 1:1 line more markedly than temperature, a linear regression line is broadly consistent with the 1:1 line.
